# Agenda setting for essential medicines policy in sub-Saharan Africa: a retrospective policy analysis using Kingdon’s multiple streams model

**DOI:** 10.1186/s12961-021-00724-y

**Published:** 2021-05-03

**Authors:** Alison T. Mhazo, Charles C. Maponga

**Affiliations:** 1 Ministry of Health, Community Health Sciences Unit (CHSU), Private Bag 65, Area 3, Lilongwe, Malawi; 2 Department of Pharmacy and Pharmaceutical Sciences, Faculty of Medicine and Health Sciences, University of Zimbabwe, P. O. Box A178, Avondale, Zimbabwe

**Keywords:** Agenda setting, Essential medicines, Policy analysis, Kingdon’s model, Primary healthcare, Universal health coverage, Sub-Saharan Africa

## Abstract

**Background:**

Lack of access to essential medicines presents a significant threat to achieving universal health coverage (UHC) in sub-Saharan Africa. Although it is acknowledged that essential medicines policies do not rise and stay on the policy agenda solely through rational deliberation and consideration of technical merits, policy theory is rarely used to direct and guide analysis to inform future policy implementation. We used Kingdon’s model to analyse agenda setting for essential medicines policy in sub-Saharan Africa during the formative phase of the primary healthcare (PHC) concept.

**Methods:**

We retrospectively analysed 49 published articles and 11 policy documents. We used selected search terms in EMBASE and MEDLINE electronic databases to identify relevant published studies. Policy documents were obtained through hand searching of selected websites. We also reviewed the timeline of essential medicines policy milestones contained in the Flagship Report, *Medicines in Health Systems: Advancing access, affordability and appropriate use*, released by WHO in 2014. Kingdon’s model was used as a lens to interpret the findings.

**Results:**

We found that unsustainable rise in drug expenditure, inequitable access to drugs and irrational use of drugs were considered as problems in the mid-1970s. As a policy response, the essential drugs concept was introduced. A window of opportunity presented when provision of essential drugs was identified as one of the eight components of PHC. During implementation, policy contradictions emerged as political and policy actors framed the problems and perceived the effectiveness of policy responses in a manner that was amenable to their own interests and objectives.

**Conclusion:**

We found that effective implementation of an essential medicines policy under PHC was constrained by prioritization of trade over public health in the politics stream, inadequate systems thinking in the policy stream and promotion of economic-oriented reforms in both the politics and policy streams. These lessons from the PHC era could prove useful in improving the approach to contemporary UHC policies.

## Background

Access to essential medicines has regained prominence as part of universal health coverage (UHC) and Sustainable Development Goals [1]. UHC is an aspiration that all individuals and communities receive the health services they need without suffering financial hardship [2]. Despite the central role of essential medicines in health systems, an estimated one third of the global population lacks access to them [3]. Medicines play a key role in fulfilling the key dimensions of UHC, namely access to quality healthcare and protection from financial hardship. In relation to quality healthcare, medicines play a critical role as curative, rehabilitative and palliative agents. Regardless of their intended use, the utilization of medicines imposes an undue financial burden at individual, household, community and national levels, particularly in low- and middle-income countries (LMICs) [4]. Although the importance of medicines can be traced back centuries, the discovery of “wonder drugs” in the mid-1940s and their dramatic promotion represents a significant milestone in pharmaceutical management [5]. The role of essential medicines in health systems has evolved tremendously, enjoying moments of favourable attention and episodes of policy uncertainty and controversy. These policy swings are driven by the interplay of institutions, ideas and interests in the political and policy domain. In turn, the maze of upstream determinants steer essential medicines policies from a technical issue requiring intellectual merit to a political issue that involves competition of interests. The way governments and institutions formulate policies bears a direct and indirect effect on the allocation of medicines in society. In turn, those policy choices can facilitate access to medicines for some groups whilst constraining access to other groups. At a global level, the geographical access to essential medicines reflects the structural determinants of inequality which raises the importance of the matter to the level of global politics [6]. This makes access to medicines a matter of public policy; an issue where policy choices have consequences on immediate and long-term status of individuals and societies. Despite the public policy nature of essential medicines and the influence of politics and power in shaping policy, public policy frameworks and policy theories are rarely applied to analyse issues in the area. This study traces the historical ascendance of essential medicines policy to the global health agenda using a public policy framework for agenda setting—the Kingdon model [7]. In this study, the terms drugs and medicines will be used interchangeably. Though the current terminology is medicines, the term drugs will be used for historical purposes.

## Aim and objectives of this study

### Aim

The aim of this study was to conduct a document and literature review on the medicines policy challenges experienced in sub-Saharan Africa during the primary healthcare (PHC) era.

### Specific objectives of the study

1. To apply Kingdon’s model to identify the contextual factors that facilitated the emergence of the essential drugs concept under PHC
2. To assess the factors that motivated the issue of access to medicines to be considered as a global problem under PHC
3. To explain how the interaction of politics and policies shaped the implementation of medicine policies in sub-Saharan Africa during PHC
4. To draw lessons and experiences from the PHC era

### Analytical framework: agenda setting using Kingdon’s model

Agenda setting refers to how a particular issue gains the attention of policy-makers amongst other issues competing for priority. Kingdon’s framework was primarily conceived to analyse public policy issues in the United States, but it has been applied for global issues, including health [8, 9]. According to Kingdon’s model, public policy is made up of three independent streams: problem stream, policy stream and politics stream. The problem stream refers to the perceptions of problems as public matters requiring intervention. The policy stream consists of the ongoing analyses of problems and their proposed solutions together with the debates surrounding these problems and possible responses. The politics stream is comprised of events such as swings of national mood, changes of government and campaigns by interest groups. Kingdon’s model recognizes the role of policy “entrepreneurs” who take advantage of agenda-setting opportunities—known as policy windows—to move items onto the formal agenda. These policy entrepreneurs can be visible or “hidden”. The visible participants are organized interest groups that highlight a specific problem, put forward a particular point of view, advocate a solution and use the mass media to draw attention to an issue of interest. Policy entrepreneurs can raise the profile of an issue during “focusing events”—a momentous event that brings an unprecedented favourable attention to an issue of public importance. The hidden participants are more likely to be the specialists in the field—the researchers, academics and consultants who work predominantly in the policy stream to develop and propose options for consideration.

## Methods

We used Kingdon’s model to frame agenda setting for essential medicines policy using a qualitative process tracing method. The process tracing method was used because it can assist in gaining insight into causal mechanisms and add an inferential advantage that is often lacking in quantitative analysis [10]. This article used elements of the scoping review methods developed by Arksey and O’Malley [11] to identify the key concepts that underpinned the agenda setting and policy formulation for medicines policy in sub-Saharan Africa. A full scoping review was not conducted because the aim of the study is not to synthesize evidence but to apply Kingdon’s model to structure and explain the underlying factors that led essential medicines policy to emerge on the global agenda and how policy formulation evolved in sub-Saharan Africa. The Arksey and O’Malley [11] framework describes five stages for conducting a scoping study: identifying the research question, identifying relevant studies, study selection, charting the data, and collating, summarizing and reporting the results.

### Identifying relevant studies

Relevant literature was obtained from a mix of hand searching and electronic database search. We hand-searched the websites of the United Nations, World Health Assembly, WHO and UNICEF. The WHO Institutional Repository for Information Sharing (IRIS) was specifically searched for key resolutions and decisions on essential drugs from the 1970s to 1990. Key health system and medicines policy events and milestones were obtained from the timeline of essential medicines policies milestones presented in the 2014 WHO Flagship Report on essential medicines [12]. A literature search on the implementation of the essential drugs policies under PHC was conducted in EMBASE and MEDLINE electronic databases using the search terms “essential” AND “drugs” AND “primary” AND “health” AND “care” for the period of 1975 to 1995. The period from the mid-1990s onwards was excluded in the literature search because of the dominance of the HIV/AIDS pandemic and the urgent need to provide medicines to avert a crisis in sub-Saharan Africa. As a result, from a methodological perspective, Kingdon’s model has limited applicability for that period since it was primarily conceived for explaining agenda setting under noncrisis situations or “politics-as-usual” circumstances [13]. Snowballing was carried out to expand the base of policy documents. We did not conduct informant interviews with actors who were involved in the formulation of the essential drug policy because of the feasibility constraints in finding key informants for a policy that was formulated nearly 50 years ago.

A literature review was selected as an appropriate method instead of a systematic review because of the nature of the review question and related study attributes [14]. The first methodological consideration was that this study presented an overview of a potentially large and diverse body of literature pertaining to a broad topic. The second consideration was that the goal of the study was not to pool evidence but to gain insight into causal mechanisms that shaped agenda setting for essential medicines within the lens of Kingdon’s model. We assumed that a period of 20 years (1975–1995) was long enough for the purposes of analysing the emergence of the essential drugs policy and sustaining of the issue on the agenda. The results of the literature review were categorized according to the components of the WHO access to essential medicine framework: sustainable financing, rational selection, affordable prices and reliable supply systems [15].

### Selecting the studies

**Fig. 1:**
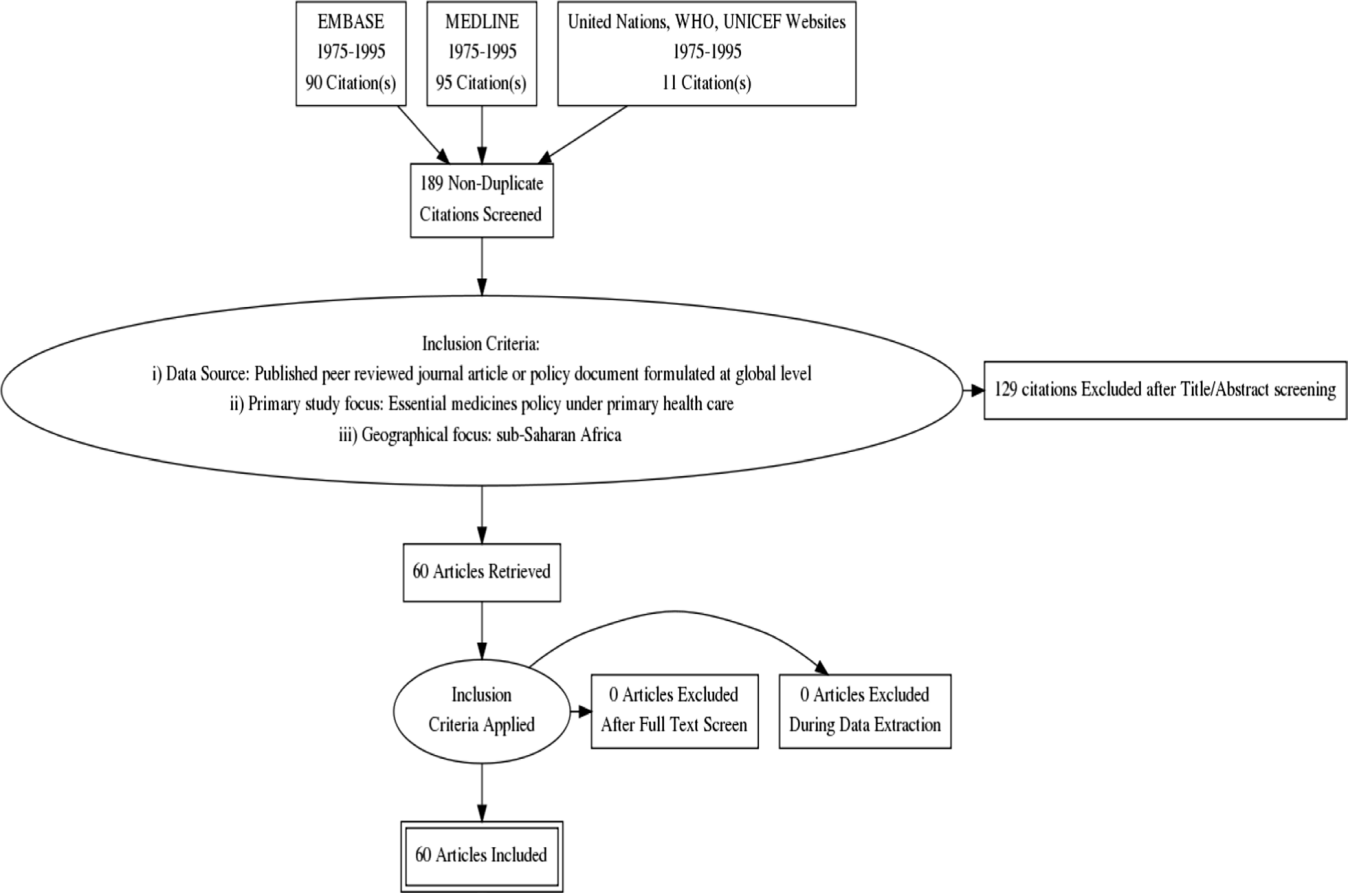
PRISMA Flow diagram for study selection: Essential medicines policy under PHC. An initial search of EMBASE and MEDLINE electronic databases generated 185 studies (90 from EMBASE and 95 from MEDLINE). Eleven (11) policy documents formulated at global level were obtained from a hand searching of United Nations, WHO and UNICEF websites making a total of 196 articles. Out of the 196 articles, 11 duplicates were removed and 189 non-duplicates were retained for further analysis. The following inclusion criteria was applied on the remaining 189 articles a) Published peer reviewed journal study or policy document formulated at global level b) Primary study focus on essential medicines policy under PHC c) Geographical focus on sub-Saharan Africa. Using this inclusion criteria, a total of 129 articles were excluded after title and abstract screening and 60 articles were retained for a further review. After full text screening, all the 60 articles (49 studies and 11 policy documents formulated at global level ) met the inclusion criteria

The Preferred Reporting Items for Systematic Reviews and Meta-Analyses (PRISMA) guidelines  were used to structure the study selection.  The PRISMA flow diagram below summarizes the databases searched, the inclusion criteria used and the number of articles reviewed (Fig. 1).

### Charting the data

Akin to data extraction, a process of data charting was conducted. The process involved creation of a Microsoft Excel master table that captured the year of publication, the title of the article, study author, publisher, the geographical origin of the study and the main study contents. The results of the literature review were categorized according to the components of the WHO access to essential medicine framework. Documents that we obtained from the hand searching of selected websites were summarized according to the organization that created the document and the name of the document or an event that created the document.

### Findings

The tables below summarize the findings from the global policy document review and literature search. Global policy document review traced how the essential drugs emerged under PHC. On the other hand, the literature search analysed how essential medicines remained a priority on the global health agenda with a particular focus on sub-Saharan Africa. Table 1 shows the results of the global policy document review organized according to the year to trace the chronology events for agenda setting for essential drugs policy. The items in italics represent key focusing events or policy windows (a favourable confluence of events that brought increased attention to drug policies). Categorization by organization gives an idea of “policy entrepreneurs” for each event. The main content highlights the politics, policy and problem issues that favoured attention to essential medicines. Table 2 summarizes the results of the literature search according to the main PHC area and specific component according to WHO’s access to medicines framework.

**Table 1:** Findings from global policy review: emergence of essential medicines policy

Year	Organization	Event/source of evidence	Main content
1974	United Nations	New economic and political order	Politics stream: increased recognition and space for expression amongst newly independent states Policy stream: global solidarity
*1974*	*WHO*	*Executive Board fifty-third session resolution*	*Problem stream: drugs not aligned to health needs* *Policy stream: Align policies with public health needs*
1975	WHO/UNICEF	Alternative approaches to meeting basic needs for developing countries	Problem stream: majority of populations lack access to health Policy stream: reorient health systems towards prevention Politics stream: ineffectiveness of Western models of care in developing countries
1975	WHO	Executive Board fifty*-*fifth session resolution	Problem stream: drugs not aligned to health needs Policy stream: align policies with public health needs
*1975*	*WHO*	*Twenty-eighth World Health Assembly resolutions*	*Problem stream: drugs not aligned to health needs, rising costs* *Policy stream: essential drugs list* *Politics stream: increased recognition of newly independent states*
*1977*	*WHO*	*Essential drugs list*	*Problem stream: proliferation of nonessential drugs* *Policy stream: limited list of drugs*
*1978*	*WHO/UNICEF*	*Primary healthcare*	*Problem stream: inadequate attention to prevention* *Policy stream: essential drugs part of PHC component* *Politics stream: global solidarity and fairness*
*1981*	*WHO*	*Action Programme on Essential Drugs*	*Problem stream: Inadequate capacity for developing countries to formulate own national drug policies* *Policy stream: Action program on essential drugs*
*1985*	*WHO*	*Conference of experts on the rational use of drugs*	*Problem stream: inappropriate use of drugs* *Policy stream: implementation of national drug policies* *Politics stream: political resistance for imposing user charges on drugs by developing countries*
1987	UNICEF	Bamako Initiative	Problem stream: increasing drug costs Policy stream: user fees for drugs and revolving fund Politics stream: structural adjustment programmes, donor fatigue
1987	WHO	Harare Declaration	Problem stream: increasing drug costs Policy stream: user fees for drugs and revolving fund Politics stream: structural adjustment programmes, donor fatigue

*In Table 1 above, italicized text indicates focusing events and policy windows for drug policy agenda setting

**Table 2:** Findings from literature search 1980–1995: sustaining essential medicines policy on the global health agenda

PHC area	Number of articles	Number of articles by specific area (in parentheses)
Selection	17	Rational selection (7), rational use (4), sustainable financing (4), reliable health and supply systems (2)
Maternal and child health	11	Reliable health and supply systems (6), sustainable financing (3), rational selection (1), rational use (1)
Disease prevention and control	7	Reliable health and supply systems (2), rational selection (2), sustainable financing (2), rational use (1)
Community access	6	Reliable health and supply systems (5),rational use (1)
Financing	4	Sustainable financing (4)
General access	2	Reliable health and supply systems (2)
Primary level access	1	Reliable health and supply systems (1)
Selective primary healthcare	1	Affordable prices (1)
Total	49	49

Specific area of the WHO access framework	Number of studies	
Reliable health and supply systems	18	
Sustainable financing	13	
Rational selection	10	
Rational use	7	
Affordable prices	1	
Total	49	

## Discussion

### Emergence of the essential drugs policy under PHC

The emergence of the essential drug policy under PHC was driven by the prevailing problems that led to remedial responses in the policy and politics systems. This section discusses the underlying factors that influenced the issue’s ascendance to the global agenda. Particular attention is drawn to how the priorities in the politics stream (expressed through international organizations) shaped the framing of the medicine problem issue, which in turn influenced the nature and content of policy responses in sub-Saharan Africa.

### Problem stream

The Twenty-eighth World Health Assembly held from the 13th to the 30th of May 1975 was an important focusing event that highlighted significant problems with global drug access [16]. WHO reported a huge disparity in drug access between developed and developing countries characterized by a higher absolute drug expenditure in developed countries and a higher proportionate expenditure in developing countries. Another key problem was that a substantial proportion of the budget was being spent on marginally effective and irrelevant drugs. As a result, large segments of the population in urgent need of essential drugs could not access them. During an era of global solidarity and fairness, the inequities were framed as a form of social injustice to the circumstances of the underprivileged. Unethical trading and commercially driven promotional practices were also identified as a problem. In that regard, developed countries were criticized for exporting drugs of questionable quality to developing countries and promoting use for unapproved purposes. The intensity of drug promotional activities in developed countries during the period also fuelled overconsumption of nonessential drugs that skewed research and development towards products with high profit potential which sidelined the urgent needs of developing countries. Developing countries also raised concern about the high cost of imported drugs with questionable quality.

### Policy stream

To respond to the problems, actors in the policy stream capitalized on policy windows to draw attention to the issue. The WHO Executive Board, at its fifty-third session, discussed the importance of prophylactic and therapeutic substances for the health of populations and the urgent need to develop drug policies linking drug research, production and distribution with real health needs [17]. This was reiterated by the fifty-fifth session of the WHO Executive Board which recommended the World Health Assembly to pay particular attention to prophylactic and therapeutic substances as a matter of major public health importance [18]. The board underscored the implementation of essential drugs programs, particularly supporting member states to develop their own national drug policies. The disproportionate expenditure on drugs that had been identified as a problem in developed countries was also seen in developing countries. To tackle the problem, the policy stream proposed policies aimed at reducing drug inflation through expenditure optimization in developing countries. The policy stream framed the problem of irrational drug use as driven by the widespread use of “nonessential drugs” driven by pharmaceutical companies’ profit motives. As a policy response, WHO put forward a proposal for countries to select a few drugs that could fulfil public health priorities. In 1977 (a year before the Alma-Ata Declaration on PHC), WHO developed the first list of essential drugs [19]. There was also a recommendation to remove trade-related constraints to public health in developing countries, including policies that allow generic manufacturing of patented drugs under compulsory licensing regimes. Realizing the relationship between effective demand for medicines and functional health systems, WHO also called for development of “sharper” health systems for medicines to align with national priorities.

### Politics stream

Although it was plagued by the Cold War politics, the 1970s has been dubbed the “warm decade for social justice” to highlight key milestones to address global social injustices [20]. During the period, decolonized African states took advantage of the United Nations “Declaration on the Establishment of a New International Economic Order” to intensify their political recognition in global governance [21]. At the heart of this movement was the need to close the socioeconomic disparities that existed between developed and developing countries in the spirit of global solidarity. Consequently, ideas that promoted social justice had a global appeal that resonated with the political environment of the day. These geopolitical developments provided a window of opportunity to push health-related issues affecting Africa to the global political agenda, particularly within the lens of decolonization and equal recognition. Access to drugs was also viewed as part of the decolonization machinery. African delegates to the Twenty-eighth World Health Assembly highlighted the problem of drug supply to liberation movements due to cancellation or mishandling of vital supplies. Resolution WHA 28.34 of the Twenty-eighth World Health Assembly called for “Activities of the WHO with regard to assistance to liberation movements in southern Africa pursuant to United Nations General Assembly Resolution 2918 (XXVII) and Economic and Social Council resolution 1804 (LV)”. Specifically, the Director-General was requested to work closely with the national liberation movements recognized by the Organization of African Unity to help identify and meet their health needs. This was reinforced by resolution 28.78, which called for WHO’s targeted assistance to newly independent and emerging states in Africa.

The politics stream had institutional and individual actors that shaped the emergence of essential drugs on the global health agenda within the PHC approach. At the institutional level, WHO and UNICEF had become critical of the health inequities that existed between the developing and developed countries [22]. In particular, there was a concern that the Western models of healthcare that focused on huge urban medical facilities did not suit the needs of the developing countries, particularly the marginalized rural population. A chief architect of this ideology was Dr Mahler, WHO Director-General who assumed the post in 1973 [23]. His ideological inclination is associated with deep religious convictions and experience working in India where he had witnessed huge urban–rural disparities in access to health. In 1975, WHO and UNICEF instituted a joint study called “alternative approaches to meeting basic health needs in developing countries” whose central theme was on highlighting the limitations of imposing Western models of health delivery to developing nations [24]. Through a series of case studies in developing countries, the study identified sufficient immunization, antenatal care, family planning, water and sanitation, health education and treatment of simple illnesses as promising approaches to address the needs of the 80% of the population that did not have access to healthcare. The studies recommended an urgent shift to an alternative model that focused on the PHC approach.

### Coupling of the streams: the Alma-Ata Declaration on primary healthcare

According to Kingdon’s model, issues do not appear on the agenda unless there is a coupling of the politics, policy and problem streams through the active participation of “policy entrepreneurs” who take advantage of policy windows and focusing events. The case history above has demonstrated how access to drugs became to be considered a problem, policy proposals that were put forward to address the problem and how the international political mood favoured advancement of such policies. Dr Mahler was a key policy entrepreneur who shaped the PHC idea, aided by his charismatic lobbying and framing of the concept within long-term aspirations of “Health for all by 2000”. In 1978, WHO and UNICEF jointly convened the PHC conference in Kazakhstan. Hundreds of delegates attended the conference, including government officials, civil society, global health institutions and public health officials. Considered a watershed moment in global health history, the delegates identified eight elements of the PHC approach that included “access to essential drugs and vaccines” in what was termed the Alma-Ata Declaration on primary healthcare [25]. The watershed event also coincided with 134 nations signing the declaration of “Health for all by 2000”. Thus, the Alma-Ata Conference was a key focusing event that brought favourable attention to PHC, including the importance of drugs.

The PHC approach was shaped by the pursuit of an alternative model that sought to “de-medicalize” provision of healthcare in favour of models that promoted disease prevention and community well-being. This goal was outlined in the joint WHO/UNICEF 1975 report titled “Alternative approaches to meeting basic needs for developing countries”. In alignment with the political discourse of the new economic order, PHC was promoted as a panacea to close the health disparities between developed and developing countries. This resonated with the international political mood that was favourable for policy reforms that carried a sense of fairness during a period of global solidarity and social justice. Soon after its emergence and widespread endorsement, PHC faced fierce resistance and a legitimacy crisis from global health actors. Critics cited feasibility constraints and inadequate funding to support such a multi-sectoral and highly ambitious approach. When Mr. James Grant became UNICEF Director in January 1980, he emerged as a counter-policy entrepreneur who advocated for an alternative approach called selective primary healthcare; an approach that focused on implementation of selected elements within the PHC framework [26]. Technically, UNICEF implemented a large-scale targeted programme aligned to its mandate in the form of growth monitoring, oral rehydration therapy, breastfeeding, immunization (GOBI) that subsequently incorporated family planning, female education and food supplementation (GOBI-FFF). Proponents of PHC resisted the selective approach citing fragmentation of health systems which directly undermined the ethos of PHC.

### Sustaining essential drugs on the agenda

The inclusion of drugs as a component of PHC brought favourable political and policy attention to the issue. Once the issue appeared on the policy agenda under PHC, there was an immediate need to move it the implementation agenda. WHO established an "Action Programme on Essential Drugs" in February 1981 in conformity with a number of resolutions of the Executive Board and the World Health Assembly [27]. In 1983, Dr Mahler showed his personal commitment to advocating the essential drugs concept by bringing the action programme directly into his own office. Whilst the problems that led to the emergence of the essential drugs concept were known, implementation required practical steps to address specific problems. In agenda-setting theory, the importance or prioritization of an issue is judged by the extent to which an issue receives coverage or attention by policy-makers, politicians, bureaucrats and researchers. In Kingdon’s model, recurrent coverage is a sign that an issue is not considered just a mere condition floating in society but a problem that requires political policy action. In this article, we singled out researchers as policy entrepreneurs and analysed the extent to which they covered the issue of drug policy in general and the specific components of the essential drug policy that received attention. We provide an analysis of the underlying drivers for such favourable attention using Kingdon’s model.

### Aspects of essential drug policy that received favourable attention

Out of the 49 studies that were reviewed between 1980 and 1995, five (5) areas of the drug policy received attention amongst the researchers: reliable health systems and supply, sustainable financing, rational selection, rational use and affordable prices. Reliable health systems and supply systems were covered in 18 studies (37%), followed by rational selection and use in 17 studies (35%) and sustainable and affordable prices in 14 studies (29%). The areas of PHC that received the most favourable attention in relation to the essential drugs policy are selection that was covered in 17 studies (35%) and maternal and child health that was covered in 11 studies (22%). The other areas that received attention in relation to medicines policy include disease prevention and control, community access, financing, general access, primary-level access and the impact of the selective primary healthcare.

### Explaining the drivers of attention: the role of problem framing

This section explains the contextual factors that favoured sustained attention to the issue of drug policy in sub-Saharan Africa after it emerged on the global agenda. We draw special attention to how the “framing” of the problem of access to drugs influenced policy choices and how dominant global ideologies favoured certain policy options ahead of others. Framing refers to “underlying structures of belief, perception and appreciation” on which distinct policy positions depend [28]. It influences the way the issue is constructed which is critical in influencing which actors get engaged in each process, the policy solutions proposed and the potential windows of opportunity that they were able to open.

### Reliable health systems and supply

The attention given to the issue of reliable health systems and supply is a reflection of the priority it was given from the agenda-setting stage. During the Twenty-eight World Health Assembly in 1975 (a focusing event that brought the issue of essential drugs to the policy agenda), WHO emphasized the need for coordinated measures to ensure a vigorous and comprehensive national effort in meeting the economic and health goals for drug policies, including the distribution of drugs. The main problem that was realized at the policy implementation stage was the weak logistics systems to deliver essential drugs to the lower levels of care, particularly in rural areas. As a result, there was inequitable distribution in favour of urban facilities. We found five (5) studies that addressed the problem of unreliable health systems and supply at the community level and two studies that were specifically designed to investigate the availability and use of drugs in rural areas [29, 30]. The attention given to access to drugs in rural areas can be understood within the underlying principles of PHC to close the rural–urban divide in access to healthcare. This ideological inclination can be traced to the WHO/UNICEF Alternatives report, a precursor to the PHC that criticized the “Western model of care” for favouring the provision of services through large urban centres at the expense of the needs of the rural population in developing countries.

### Influence of global organizations

In the politics stream, global organizations used their international experience to influence the design of supply chain systems in sub-Saharan Africa. To address the challenges of drug supply systems at lower levels of care, in the early 1980s, UNICEF promoted the distribution of preassembled hospital kits with drugs calculated based on the catchment population and the burden of disease based on the experience of the United Nations High Commissioner for Refugees (UNHCR) in refugee settings [31]. Countries that adopted this model include Kenya, Somalia and Ghana [32–34]. Though the model was once described as “state of the art” in addressing the problem of unreliable health systems and supplies, misalignment of drug kit contents with the needs of intended communities, stock outs and misallocation of drugs at the primary healthcare level led to the discontinuation of the distribution model.

The interest of international donors was not only in redesigning supply chain systems to meet the general needs but also to achieve their own objectives within their core mandate. Overall, we found that 11 studies focused on provision of essential drugs in relation to maternal and child health (infant mortality, maternal mortality and family planning) with 10 of those studies conducted between 1985 and 1995. UNICEF promoted the design of supply chain systems and provision of drugs to meet the needs of children, an approach that can be associated with their preference for a selective primary healthcare approach and implementation of the GOBI-FFF strategy. We also found that the problems in drug access started to be associated with “avoidable” maternal deaths. Whilst infant mortality had long been recognized as a problem from the 1970s to 1980s due to advances in statistical techniques and household surveys, the full extent of the problem of maternal deaths was largely unacknowledged [35]. The problem was brought to the policy agenda after a WHO 1985 report that announced that half a million maternal deaths were occurring each year, 99% of them in developing countries. In Kingdon’s terms, the study revealed a “crisis” which transformed the status of maternal mortality in developing countries from a nonserious issue to a problem that required urgent policy and political attention. The confluence of the problem, politics and policy gave birth to the Safe Motherhood Initiative (SMI) in 1985 whose implementation required the availability of drugs. The United Nations Population Fund (UNFPA, a policy entrepreneur that funded the WHO study) took advantage of the window of opportunity and framed access to essential drugs and family planning supplies within the broader context of socioeconomic development which brought favourable attention to the issue [36, 37].

### Sustainable financing and affordable prices: the problem of unsustainable expenditure

Thirteen (13) articles addressed the issue of sustainable financing for drugs. Out of those, 11 cited the problem of unsustainable drug expenditure that ranged from 25 to 50% in some developing countries; a situation that was further compounded by economic recession and associated austerity measures [38, 39]. The problem of drug financing can be traced back to the origins of the essential drugs concept in the late 1970s during the agenda-setting stage. Five (5) studies highlighted that the problem of unsustainable expenditures was driven by the profit motives of transnational pharmaceutical companies due to heavy marketing of nonessential medicines and misleading promotional claims. Two studies highlighted that professional resistance by doctors and pharmacists to implement the essential drug programme influenced the rise in drug expenditure. These problems reveal conflicting interests between the aspects of trade, personal income and public health dating back to the conception of the essential drugs concept. When the essential drugs concept was introduced in the policy stream, it suffered an immediate legitimacy crisis as pharmaceutical companies and medical associations perceived a threat to their profits and framed the idea as a threat to choice and a barrier to access [40]. This conflict can be traced back to the 1948 World Health Assembly that established WHO. During that meeting, some delegations called for WHO to provide essential medical supplies to countries that did not produce these commodities, while other delegations maintained that medicines should be handled as other commodities and obtained through “the normal peacetime economic machinery” [41, 42]. To manage the inherent tensions, up until the mid-1970s, WHO maintained a restricted mandate to provide drugs such as chloroquine, penicillin and streptomycin through vertical programmes, leaving the rest of the drugs to the functions of free markets [43]. Thus, the essential drugs concept threatened the viability of drug markets and was met with an immediate dissenting opinion from pharmaceutical companies which maintained that there was no such thing as an “inessential” drug, and only agreed to cooperate with the programme after clear assurances that the concept would be limited to the public sector of developing countries. In 1985, the United States senate made a decision to withhold its contribution to WHO’s regular budget, in part as a protest against WHO’s “essential drug” programme. Leading US-based pharmaceutical companies opposed the programme at a time when the United States was home to 11 out of 18 top pharmaceutical manufacturers [43].

### Influence of international organizations on drug financing policies: the Bamako Initiative

In 1984 the World Health Assembly requested that the WHO Director-General host a multi-stakeholder conference to discuss ways to address the problem of irrational drugs, particularly in developing countries, which resulted in the Nairobi Conference of Experts on the Rational Use of Drugs in 1985 [44]. The conference was a major focusing event that resulted in WHO’s revised drug strategy by putting emphasis beyond drug selection to encompass procurement, distribution, rational use and quality assurance. During the 1985 Nairobi Conference, the problem of drug financing was debated. Delegates from Western countries advocated for payment of drugs at the point of consumption based on a partial or full cost recovery model, whilst delegates from developing countries anticipated public backlash and political risks associated with the proposals. In the politics stream, the idea of paying for drugs was capitalistic and incompatible with the socialist model that was being pursued by newly decolonized states as they tried to dismantle the colonial legacy of social segregation. Despite the political discomfort, in September 1987 at the thirty-seventh session of the WHO Regional Committee for Africa, held in Mali, the executive director of UNICEF introduced the “Bamako Initiative: Women's and Children's Health through the Funding and Management of Essential Drugs at Community Level”. Among some major policy proposals in the policy stream, the initiative proposed user fees for drugs and establishment of drug revolving funds [39]. The Bamako Initiative deserves special attention because it is one of the few policies that attempted to solve a complex health system problem (unsustainable expenditure) through some demand-side interventions in the form of user fees.

The launch of the Bamako Initiative coincided with the dominance of market-oriented structural reforms promoted by the World Bank and the International Monetary Fund (IMF) to improve efficiencies within the social services, including the health sector. Economic-oriented institutions framed the problem of rising drug budgets in sub-Saharan Africa as an unsustainable fiscal burden, a view that was favourable during a period of deep economic pain induced by the global recession. Confronted with donor fatigue, there was also a prevailing notion that the free provision of health services was unsustainable, particularly for recurring costs such as for drugs. As a policy remedy, user fees were introduced to generate extra revenue. Although an option for user fee exemption was in-built in the design of the initiative to promote equitable access, the Bamako Initiative was a key health system milestone that determined access to drugs based on the ability to pay, departing form the norm of provision based on need. Political actors in developing countries accepted the ideas against their socialist ideologies and introduced user fees as part of wider structural adjustment programmes. Their hesitance was countervailed by an argument that individuals were already paying for drugs in the private sector at a higher price; a premise that conflated willingness to pay with the ability to pay in the policy stream. In UHC terms, the new policy did not fully consider the adverse implications of user fees from an equity and efficiency point of view. The equity dimension is that individuals could sacrifice to pay for the drugs but end up in financial hardship. The efficiency dimension is that user fees can potentially force the population to forgo essential and cost-effective care at the primary level and end up with more serious conditions that require high-intensity care at higher facilities—an inefficient cost-shifting phenomenon known as the “squeezed balloon effect” [45].

The idea itself was a subject of controversy and contestation at a global level. The Director-General Dr Mahler feared that the coupling of prescribing volume to provider salaries would fuel overprescribing and was opposed to the idea [46]. To illustrate the role of policy entrepreneurs, when Dr Nakajima took over from Dr Mahler as WHO Director-General in 1988, he did not sustain the resistance. Instead, one of his first acts on becoming director-general was to move the action programme on essential drugs out of his office into its own division in a sign of the diminishing status of the programme.

A multi-country evaluation of the Bamako Initiative for Burundi, Guinea, Kenya, Uganda and Nigeria reported various challenges. The evaluation study showed that in all the countries, facility staff manipulated drug-pricing structures to maximize their own income [47]. Polypharmacy and irrational drug use persisted in Nigeria where the pricing regime was based on the volume of drugs prescribed, whilst in Guinea, a system of cost recovery linked to diagnosis induced multiple diagnoses. In Kenya, there was resistance to prescribe subsidized products that did not guarantee a monetary incentive. Typically, these challenges present a bigger challenge of supplier-induced demand where providers stimulated unnecessary consumption to maximize their own revenue, exacerbating the very same inefficiencies meant to be addressed by the initiative. From a public policy and implementation science perspective, the health workers’ strategic reaction to a set of “top-down” interventions is an example of an emergent behaviour and negative feedback loop in complex systems. This is when well-intended reforms induce an unexpected behaviour amongst service providers or the “street-level bureaucrats” [48, 49].

### The problem of irrational drug use

Out of the 49 articles, 17 focused on the rational selection and use. During the agenda-setting stage, the problem of irrational selection prompted the policy stream to introduce the essential drugs concept and the subsequent development of the first WHO model list in 1977. At the policy formulation stage, the Nairobi Conference further brought the issue to policy attention. In the policy stream, priority was given to the training of prescribers on rational drug use and adaptation of global guidelines to suit local contexts to tackle the problem of rational drug use. Building on the Nairobi Conference momentum, in 1989, the policy stream responded to the problem of irrational drug use through the formation of the International Network for Rational Use of Drugs (INRUD) [50]. INRUD was created to solve the problem of irrational drug use through a coordinated approach between groups of researchers from four African and three Asian countries with support groups in Boston, Sweden, WHO and Australia. The activities of INRUD were supported by multilateral, bilateral and foundation donors and by Management Sciences for Health (MSH).

## Limitations of the study

This study applied Kingdon’s theory to analyse agenda-setting essential medicine policy to explain how key contextual factors facilitated the ascendancy of the issue to the global health agenda and its subsequent implementation in sub-Saharan Africa. However, there are some limitations worth mentioning. The first one is that we did not conduct informant interviews with actors who were involved in the formulation of the essential drug policy. This is because of the feasibility constraints in finding key informants for a policy that was formulated nearly 50 years ago. The other limitation is that we did not conduct a comprehensive systematic review because the subject is so broad, with a heterogeneous study design and aims.

## Conclusion

Policy theories are useful to direct and guide analysis, deepen understanding and provide explanations for the formulation and implementation of medicine policies in sub-Saharan Africa. Using Kingdon’s model as a lens for interpretive analysis, we found that the essential medicines policy emerged under primary healthcare in response to the problems of unsustainable rise in medicine expenditure, pervasive inequities in global access to medicines and widespread irrational use. During implementation, actors in the politics and policy streams strategically shaped the framing of these problems to exert policy choices on drug selection, financing and use. We found that effective implementation of medicines policies under PHC was constrained by the prioritization of trade over public health in the political stream, inadequate systems thinking in the policy stream and promotion of economic-oriented reforms in both the politics and policy streams. These lessons from the PHC era could prove useful in improving the approach to medicines policies, for example, under the contemporary UHC discourse.

## Data Availability

The data sets used and/or analysed during the current study are available from the corresponding author on reasonable request.
